# Correlation between ZJU index and hepatic steatosis and liver fibrosis in American adults with NAFLD

**DOI:** 10.3389/fmed.2024.1443811

**Published:** 2024-08-15

**Authors:** Shuang Luo, Xiaolu Weng, Jing Xu, Hao Lin

**Affiliations:** ^1^Department of Gastroenterology, Pingyang Hospital of Wenzhou Medical University, Wenzhou, China; ^2^Department of Endocrinology, The Second Affiliated Hospital and Yuying Children's Hospital of Wenzhou Medical University, Wenzhou, China

**Keywords:** ZJU index, NAFLD, hepatic steatosis, liver fibrosis, obesity

## Abstract

**Background:**

ZJU index, a novel calculation combining blood glucose, body mass index (BMI), lipids and liver functions, is closely related with non-alcoholic fatty liver disease (NAFLD). However, the correlation between ZJU index and hepatic steatosis and liver fibrosis has not been reported in the studies. This study aims to examine the correlation between these variables.

**Methods:**

Data from the 2017–2020 NHANES were collected for a cross-sectional study, to explore the linear relationship between ZJU, liver stiffness measurements (LSM) and controlled attenuation parameters (CAP) with multivariate linear regression models. Restricted cubic spline (RCS) regression and threshold effect analyses were utilized to describe the nonlinear relationship. The correlation in subgroups was analyzed based on race, gender, drinking, age, BMI, diabetes and moderate activities.

**Results:**

In this population-based study, a total of 2,122 adults aged 18–80 years old with NAFLD were included. According to the multivariate linear regression analysis, ZJU had a significant positive correlation with liver fibrosis (LSM, *β* = 0.182, 95%CI = 0.154–0.211, *p* < 0.001) and hepatic steatosis (CAP, *β* = 2.35, 95%CI = 2.14–2.56, *p* < 0.001), which was stronger in males. According to the RCS analysis, an inverted L-shaped relationship between ZJU and CAP (inflection point at 60.56) and a J-shaped relationship between ZJU index and LSM (inflection point at 51.27) were observed.

**Conclusion:**

ZJU had a positive correlation with CAP and LSM in American adults with NAFLD. The findings suggest that ZJU may be a valuable biomarker for assessing the severity of liver fibrosis and hepatic steatosis in individuals with NAFLD.

## Introduction

Non-alcoholic fatty liver disease (NAFLD) is recognized as the primary causative factor for chronic liver diseases, affecting approximately up to 25% among the population (1). Simultaneously, as the rates of metabolic syndrome and obesity continue to rise, the occurrence of NAFLD is also increasing worldwide. The accumulation of excessive fat in liver not only can result in local effects such as dysfunction of hepatocytes, fibrogenesis and activation of proinflammatory immune responses, but also can contribute to the development of various extrahepatic metabolic disorders, including type 2 diabetes mellitus (T2DM) and cardiovascular events (2). Additionally, observational studies have demonstrated a correlation between liver fibrosis, steatosis, and an elevated risk of all-cause mortality. Hence, the assessment on liver fibrosis and steatosis levels is essential for the evaluation and clinical prognosis of individuals with NAFLD (3).

Liver biopsy is traditionally considered as the gold standard for assessing the severity of liver fibrosis and steatosis. Vibration controlled transient elastography (VCTE) is emerging as a non-invasive alternative. VCTE can measure two key parameters, liver stiffness measurements (LSM) and controlled attenuation parameters (CAP), and recent observational studies have indicated its strong accuracy in predicting the degree of steatosis and fibrosis stage in liver (4, 5). However, VCTE is constrained by its high learning curve and popularity when using (6).

ZJU index, a novel metabolic parameter incorporating changes in various indicators such as aspartate aminotransferase (AST), triglyceride (TG), alanine aminotransferase (ALT), BMI and blood glucose (7), can coffer a more holistic perspective and effectively capture trends in metabolic disorders and associated disease risks compared to individual biochemical markers. Studies have indicated its correlation with lipid metabolism, insulin resistance (IR) and obesity, with initial development focusing on predicting NAFLD and subsequent validation demonstrating its strong predictive capacity (7, 8).

However, there has been a lack of research on the correlation between ZJU index and CAP and LSM. The studies on the interrelationship among these variables may provide innovative perspectives for monitoring the severity of NAFLD, utilizing only lipids, blood glucose, liver function, and anthropometric measurements. ZJU index has its potential to serve as a convenient and cost-effective tool for evaluating the disease. Consequently, the current study was conducted.

## Materials and methods

### Data and sample sources

The data included in this investigation were sourced from the 2017–2020 NHANES, encompassing a diverse, representative sample of the non-institutionalized population in the United States. These cross-sectional surveys were administered by NCHS.

**Figure 1:**
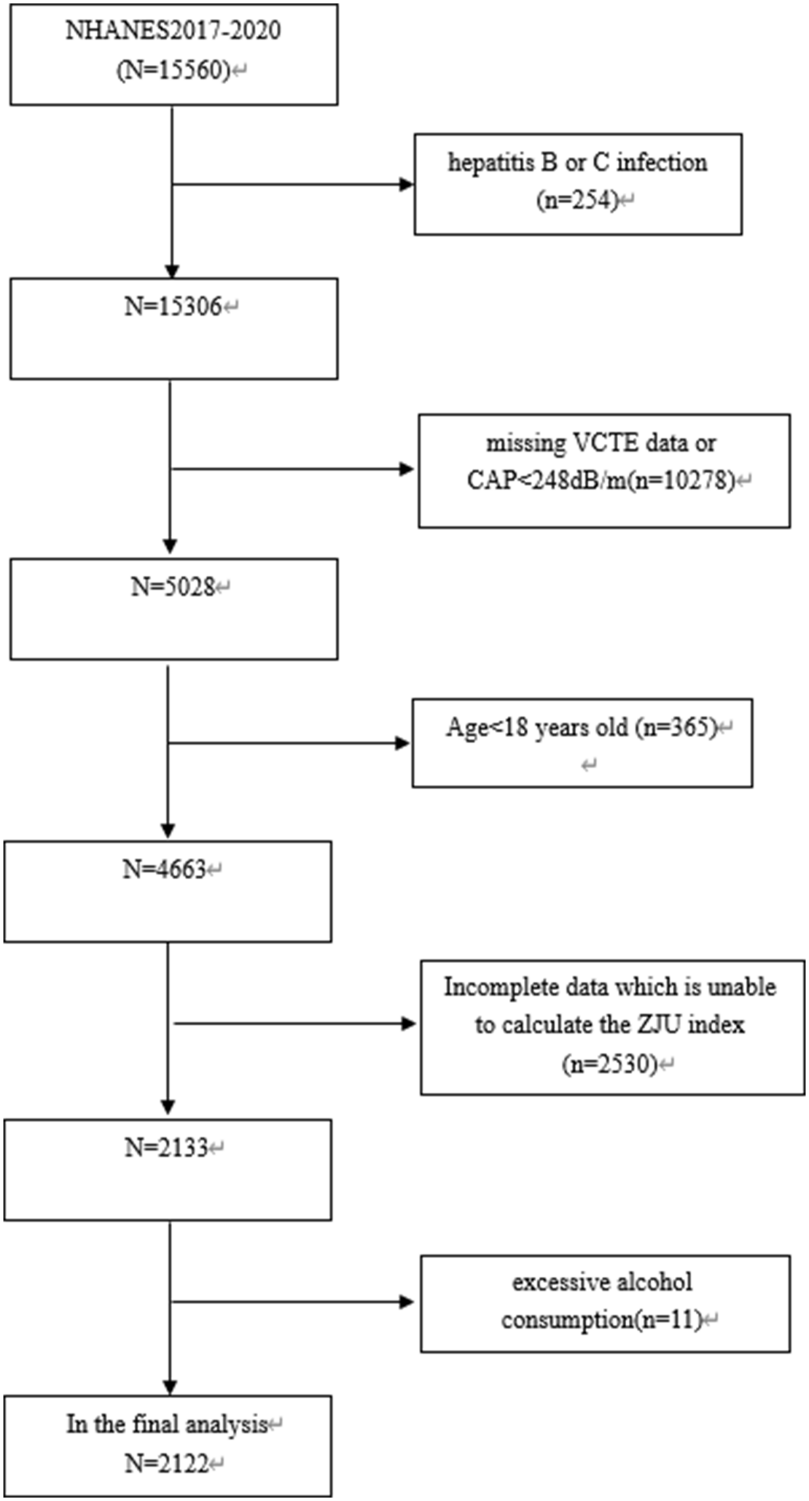
Flowchart of the sample selection from the 2017–2020 NHANES.

Among 15,560 subjects, those with hepatitis C or B infection (*n* = 254), those missing VCTE data or CAP<248 dB/m (*n* = 10,278), those under 18 years old (*n* = 365), those with incomplete data preventing calculation of ZJU index (*n* = 2,530), and those with excessive alcohol consumption (defined as consuming 4/5 or more drinks daily) (*n* = 11) were excluded. Finally, 2,122 subjects were included in this study (as shown in Figure 1).

The implementation of NHANES was approved by the Ethics Review Board of NCHS, and all subjects have provided written informed consent (9).

### Definition of ZJU index

ZJU index = FPG (mmol/L) + BMI (kg/m^2^) +3*ALT(U/L)/AST(U/L) ratio (+2 if female) + TG (mmol/L) (7).

### Vibration controlled transient elastography (VCTE)

In the database of NHANES, VCTE of liver measured in subjects from 2017 to 2020 with the FibroScan 502 V2 Touch (Echosens), was well-suited for NAFLD. In order to assess liver fibrosis and steatosis in patients with NAFLD, validated parameters such as LSM and CAP were employed (10, 11). For VCTE outcomes to be considered valid, certain criteria were followed, including acquiring a minimum of 10 LSMs after fasting for at least 3 h, with an interquartile range (IQR) /median of less than 30% (12). It is used to determine that patients with CAP ≥248 dB/m had NAFLD (13).

### Covariates

Through a comprehensive examination on existing literature, potential confounding covariates were identified in the multivariable-adjusted model for the relationship between ZJU and hepatic steatosis and liver fibrosis (14–17). Demographic covariates in the study encompassed race, age and gender, while anthropometric and laboratory covariates included ALT, BMI, weight, total cholesterol (TC), waist circumference, albumin, gamma-glutamyl transpeptidase (GGT), creatinine, alkaline phosphatase (ALP), low density lipoprotein cholesterol (LDL-C), FPG, high density lipoprotein cholesterol (HDL-C), total bilirubin, uric acid, and TG. Medical history covariates included the presence or absence of hypertension and T2DM, moderate physical activities, and alcohol consumption.

### Statistical analysis

In accordance with the requirements of NCHS, all the analyses in this study were conducted with complex sampling design and sample weights. The following formula was adopted to calculate the sample weight: Sample weight = 1/2*two-year subsample weight (years in 2017–2018), or 1/2*two-year subsample weight (years in 2019–2020). Differences between ZJU index quartiles were assessed through weighted Student’s t-tests for continuous variables and weighted chi-squared tests for categorical variables. The correlation between ZJU index and CAP and LSM were examined with three weighted multivariable linear regression models. In Model 1, any of covariates was not adjusted, while in Model 2, adjustments were made for race, age and gender. In Model 3, adjustments were made for race, age, gender, diabetes mellitus, moderate activities, hypertension, drinking, albumin, uric acid, GGT, ALP, total bilirubin and creatinine.

Smooth curve fitting utilizing restricted cubic splines (RCS) was employed to assess the nonlinear correlation between ZJU and CAP, LSM. In cases of non-linear correlation, a two-piecewise linear regression model was adopted to fit each interval and determine the threshold effect. The inflection point (K) was identified with a two-step recursive method. Additionally, a subgroup analysis was conducted to examine the correlation between ZJU index and CAP and LSM. In this analysis, stratified multivariable linear regression models with covariates were utilized, including age, gender, T2DM, BMI, hypertension, alcohol consumption and moderate activities. The analysis was performed with R (version 4.1.3) and Free Statistics software (version 1.9.2). Statistical significance was determined as a two-sided *p* ≤ 0.05.

## Results

### Characteristics of subjects

In this study, a total of 2,122 subjects aged 18 years old or more, with the mean age of 52.23 years old, were included, among which, 51.3% of the subjects were males and 48.7% were females. ZJU index was divided into quartiles ranging as Q1:<39.12, Q2:39.12–43.61, Q3:43.61–49.72, Q4:>49.72, respectively. The subjects were stratified into four groups according to ZJU quartiles, revealing notable variations in race, gender, diabetes mellitus, moderate physical activity and hypertension (all *p* < 0.001). Among the quartiles, the subjects in Quartile 4 had elevated BMI, waist circumference, ALT, GGT, uric acid, FPG, TG levels, as well as a higher incidence of hypertension and diabetes mellitus compared to those in other groups. Particularly noteworthy, the subjects in Quartile 4 displayed higher values for LSM and CAP. Further detailed information can be found in Table 1.

**Table 1:** Weighted characteristics of the study population based on ZJU quartiles.

Characteristic	Q1 (<39.12)	Q2 (39.12–43.61)	Q3 (43.61–49.72)	Q4 (≥49.72)	*p* value
Number	530	531	531	530	
Age, year	53.9 ± 17.7	53.8 ± 16.1	52.2 ± 16.8	49.0 ± 15.5	<0.001
Sex, %					<0.001
Male	63.4	55.7	50.8	35.3	
Female	36.6	44.3	49.2	64.7	
Race, %					<0.001
Mexican American	11.7	14.5	19.2	16.8	
Other Hispanic	8.7	12.4	12.4	9.1	
Non-Hispanic White	40.0	36.9	32.6	35.5	
Non-Hispanic Black	15.1	15.4	22.8	29.6	
Other Race	24.5	20.7	13.0	9.1	
Moderate activities, %					<0.001
Yes	47.0	40.9	40.1	34.2	
No	53.0	59.1	59.9	65.8	
Diabetes, %					<0.001
Yes	9.1	17.7	22.4	35.5	
No	80.9	82.3	77.6	64.5	
Hypertension, %					<0.001
Yes	34.3	42.0	47.1	56.4	
No	65.7	58.0	52.9	43.6	
Drinking, %					0.600
Current or ever	86.0	85.3	87.2	87.9	
Never	14.0	14.7	12.8	12.1	
Weight, kg	72.2 ± 12.2	82.9 ± 13.1	94.5 ± 15.2	115.3 ± 23.9	<0.001
BMI, kg/m^2^	25.2 ± 2.4	29.5 ± 2.1	33.6 ± 2.9	41.9 ± 7.2	<0.001
Waist circumference, cm	92.1 ± 8.9	102.2 ± 8.2	110.8 ± 9.5	125.1 ± 13.9	<0.001
Albumin, g/dl	4.12 ± 0.31	4.05 ± 0.30	3.98 ± 0.30	3.83 ± 0.31	<0.001
ALT, U/L	20.1 ± 13.1	24.5 ± 16.7	26.7 ± 17.8	27.2 ± 20.6	<0.001
AST, U/L	22.7 ± 17.1	21.9 ± 10.8	22.3 ± 11.8	21.4 ± 11.4	0.148
GGT, U/L	31.4 ± 46.5	34.7 ± 39.0	35.4 ± 37.3	37.3 ± 41.6	0.030
ALP, U/L	78.0 ± 28.2	78.9 ± 22.7	78.4 ± 21.6	86.6 ± 29.2	<0.001
Total bilirubin, mmol/L	(9.0 ± 5.2)^*^10^−3^	(8.5 ± 4.7)^*^10^−3^	(8.1 ± 4.7)^*^10^−3^	(7.3 ± 4.0)^*^10^−3^	<0.001
Creatinine, mmol/L	82.1 ± 52.3	78.3 ± 31.9	79.4 ± 48.8	72.7 ± 22.9	<0.001
Uric acid, mmol/L	0.323 ± 0.080	0.340 ± 0.085	0.348 ± 0.084	0.352 ± 0.093	<0.001
FPG, mmol/L	5.8 ± 0.8	6.2 ± 1.2	6.6 ± 1.9	8.1 ± 3.8	<0.001
TC, mmol/L, mmol/L	4.90 ± 1.07	4.90 ± 1.07	4.76 ± 1.06	4.73 ± 1.07	0.012
TG, mmol/L	1.29 ± 0.65	1.55 ± 0.89	1.71 ± 0.98	1.94 ± 1.57	<0.001
LDL-C, mmol/L	2.88 ± 0.93	2.95 ± 0.93	2.82 ± 0.92	2.81 ± 0.94	0.176
HDL-C, mmol/L	1.49 ± 0.47	1.30 ± 0.34	1.23 ± 0.33	1.17 ± 0.31	<0.001
CAP, dB/m	283.3 ± 29.6	299.2 ± 34.9	313.2 ± 38.9	333.9 ± 44.0	<0.001
LSM, kPa	5.32 ± 4.44	5.69 ± 3.31	6.19 ± 4.01	8.65 ± 7.67	<0.001

Values are mean ± SD or number (%). P < 0.05 was deemed significant. BMI, body mass index; TC, total cholesterol; TG, triglyceride; HDL-c, High density lipoprotein cholesterol; LDL-c, Low density lipoprotein cholesterol; ALT, alanine aminotransferase; AST, aspartate aminotransferase; GGT, gamma-glutamyl transpeptidase; ALP, alkaline phosphatase; LSM, liver stiffness measurements; CAP, controlled attenuation parameter.

### Correlation between ZJU index and hepatic steatosis (CAP)

Initially, the relationship between ZJU and the severity of liver steatosis was assessed without controlling for covariates. A positive correlation was observed between higher ZJU index and increased hepatic steatosis grade. Following comprehensive adjustments as outlined in the section Methods, each incremental unit of ZJU index was linked to a rise of 2.35 dB/m in CAP units [*β* = 2.35, 95% CI (2.14, 2.56), *p* < 0.001]. Sensitivity analysis was performed by categorizing ZJU index into quartiles. In the totally adjusted model, β coefficients of ZJU index for subjects in the second, third, and fourth quartiles were found to be 15.33, 29.72, and 52.72, respectively, compared to the lowest quartile (the first quartile) as indicated in Table 2.

**Table 2:** The associations between ZJU with CAP and LSM value in linear regression analysis.

ZJU	Model 1	Model 2	Model 3
CAP β (95%CI)	2.21 (2.02, 2.39), <0.001	2.44 (2.26, 2.62), <0.001	2.35 (2.14, 2.56), <0.001
ZJU (Quartile)			
Q1	Reference	Reference	Reference
Q2	15.91 (11.42, 20.39), <0.001	17.11 (12.75, 21.48), <0.001	15.33 (10.97, 19.72), <0.001
Q3	29.95 (25.46, 34.43), <0.001	32.18 (27.79, 36.58), <0.001	29.72 (25.19, 34.26), <0.001
Q4	50.66 (46.17, 55.14), <0.001	56.32 (51.83, 60.80), <0.001	52.72 (47.71, 57.74), <0.001
P for trend	<0.001	<0.001	<0.001
LSM β (95%CI)	0.190 (0.166, 0.215), <0.001	0.211 (0.186, 0.236), <0.001	0.182 (0.154, 0.211), <0.001
ZJU (Quartile)			
Q1	Reference	Reference	Reference
Q2	0.375 (−0.243, 0.994), 0.234	0.486 (−0.129, 0.101), 0.121	0.180 (−0.433, 0.792), 0.566
Q3	0.875 (0.257, 1.494), 0.006	1.079 (0.460, 1.698), 0.001	0.569 (0.064, 1.202), 0.048
Q4	3.335 (2.716, 3.954), <0.001	3.794 (3.162, 4.426), <0.001	2.771 (2.072, 3.470), <0.001
P for trend	<0.001	<0.001	<0.001

Model I: None covariates were adjusted; Model II: gender, age and race were adjusted; Model III: gender, age, race, diabetes, Moderate activities, hypertension, drinking, albumin, uric acid, GGT, ALP, total bilirubin, creatinine.

### Correlation between ZJU index and liver fibrosis (LSM)

As shown in Table 2, the analysis revealed that for each unit increase in ZJU index, there was a corresponding increase of 0.182 kPa in LSM after adjusting all covariates [*β* = 0.182, 95% CI (0.154, 0.211), *p* < 0.0001]. Furthermore, adjusted β coefficients for subjects in the second, third, and fourth quartiles were 0.180, 0.569, and 2.771, respectively, compared to the lowest quartile.

### Subgroup analysis

A stratified weighted multivariate regression analysis was conducted to explore the relationship between ZJU index and CAP and LSM across various population subgroups, stratified by race, gender, BMI, age, T2DM, hypertension, drinking, and moderate physical activities. As presented in Table 3, the findings revealed a stronger positive correlation between ZJU index and CAP in male subjects and Mexican Americans (*p* < 0.05). Additionally, a stronger significant correlation between ZJU index and LSM was found in males and those with BMI > 30 kg/m^2^ (Table 4).

**Table 3:** Association between ZJU and CAP stratified by gender, age, race, diabetes, moderate activities and BMI.

	β (95%CI) p value	*P* for interaction
Stratified by gender		0.004
Male	2.64 (2.33–2.95), <0.001	
Female	2.08 (1.80–2.35), <0.001	
Stratified by race		<0.001
Mexican American	3.03 (2.49, 3.58), <0.001	
Other Hispanic	2.81 (2.06, 3.57), 0.009	
Non-Hispanic White	2.64 (2.28, 2.99), <0.001	
Non-Hispanic Black	1.76 (1.33, 2.18), 0.016	
Other Race	2.32 (1.81, 2.84), <0.001	
Stratified by age		0.165
Age < 50 years old	2.11 (1.81, 2.40), <0.001	
Age ≥ 50 years old	2.44 (2.15, 2.73), <0.001	
Stratified by BMI		0.235
BMI < 30 kg/m^2^	2.34 (1.83, 2.84), <0.001	
BMI ≥ 30 kg/m^2^	2.32 (2.00, 2.65), <0.001	
Stratified by diabetes		0.874
Yes	2.50 (2.05, 2.95), <0.001	
No	2.31 (2.06, 2.56), <0.001	
Stratified by hypertension		0.677
Yes	2.35 (2.04, 2.67), <0.001	
No	2.33 (2.05, 2.61), <0.001	
Stratified by drinking		0.985
Yes	2.34 (2.11, 2.56), <0.001	
No	2.34 (1.73, 2.94), <0.001	
Stratified by Moderate activities		0.250
Yes	2.21 (1.88, 2.55), <0.001	
No	2.41 (2.15, 2.68), <0.001	

Gender, age, BMI, race, moderate activities, diabetes, hypertension, drinking (not adjusted for in the subgroup analyses), albumin, uric acid, GGT, ALP, total bilirubin and creatinine were adjusted.

**Table 4:** Association between ZJU and LSM stratified by gender, age, race, diabetes, moderate activities and BMI.

	*β* (95%CI) *p* value	*P* for interaction
Stratified by gender		0.012
Male	0.238 (0.190, 0.286), <0.001	
Female	0.138 (0.105, 0.171), <0.001	
Stratified by race		0.219
Mexican American	0.131 (0.066, 0.196), <0.001	
Other Hispanic	0.172 (0.111, 0.233), <0.001	
Non-Hispanic White	0.197 (0.137, 0.258), <0.001	
Non-Hispanic Black	0.174 (0.117, 0.232), <0.001	
Other Race	0.202 (0.139, 0.264), <0.001	
Stratified by age		0.054
Age < 50 years old	0.228 (0.186, 0.271), <0.001	
Age ≥ 50 years old	0.135 (0.097, 0.174), <0.001	
Stratified by BMI		0.002
BMI < 30 kg/m^2^	0.047 (−0.017, 0.111), 0.149	
BMI ≥ 30 kg/m^2^	0.274 (0.228, 0.320), <0.001	
Stratified by diabetes		0.164
Yes	0.185 (0.119, 0.251), <0.001	
No	0.194 (0.159, 0.228), <0.001	
Stratified by hypertension		0.545
Yes	0.217 (0.170, 0.263), <0.001	
No	0.153 (0.117, 0.190), <0.001	
Stratified by drinking		0.614
Yes	0.185 (0.154, 0.216), <0.001	
No	0.182 (0.112, 0.253), <0.001	
Stratified by Moderate activities		0.229
Yes	0.157 (0.111, 0.203), <0.001	
No	0.200 (0.164, 0.237), <0.001	

Gender, age, BMI, race, moderate activities, diabetes, hypertension, drinking (not adjusted for in the subgroup analyses), albumin, uric acid, GGT, ALP, total bilirubin and creatinine were adjusted.

### Non-linear relationship between ZJU index and liver fibrosis and hepatic steatosis

**Figure 2:**
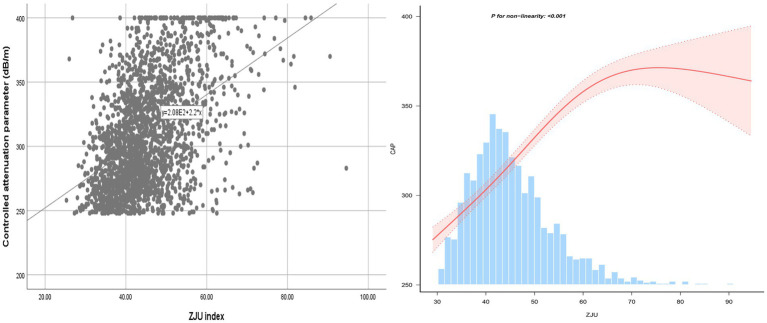
The smooth curve fit for the association between ZJU index and CAP.

Following the adjustments for all variables, a non-linear relationship between ZJU index and CAP was identified, as illustrated in Figure 2. Specifically, an inverted-L shaped correlation between ZJU index and CAP was observed, with an inflection point at 60.56. When ZJU index was below 60.56, there was a significant effect value of 2.881, while when ZJU index exceeded 60.56, the effect value was not statistically significant (Table 5).

**Table 5:** Threshold effect analysis of ZJU on CAP and LSM using the two-piecewise linear regression model.

ZJU	CAP adjusted *β* (95%CI) *p* value	LSM adjusted *β* (95%CI) *p* value
Fitting by the standard linear model	2.505 (2.283, 2.728), <0.001	0.153 (0.128, 0.178), <0.001
Fitting by the two-piecewise linear model		
Inflection point	60.56	51.27
<K segment effect	2.881 (2.625, 3.137), <0.001	0.060 (0.024, 0.096), 0.001
>K segment effect	0.389 (−0.379, 1.157), 0.321	0.296 (0.249, 0.344), <0.001
Log likelihood ratio	<0.001	<0.001

**Figure 3:**
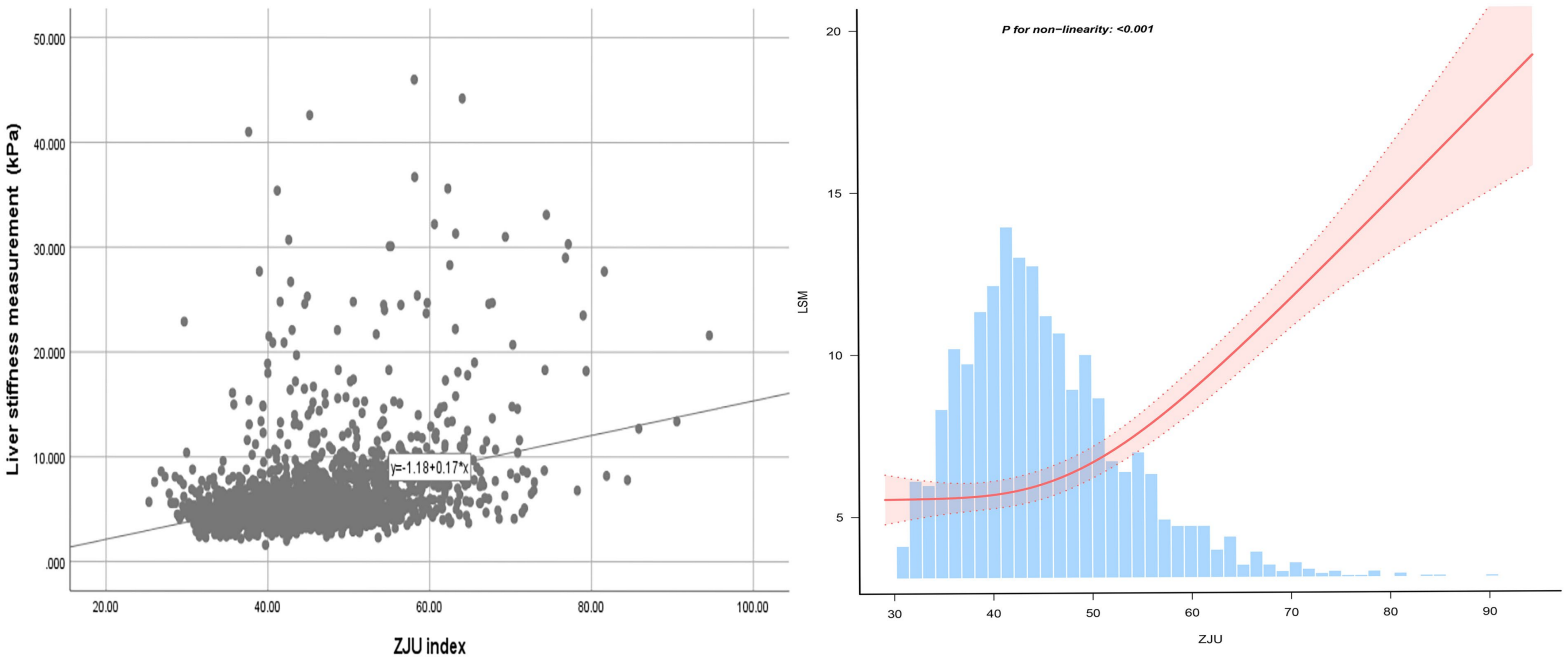
The smooth curve fit for the association between ZJU index and LSM.

A J-shaped between ZJU index and LSM when conducting the nonlinear model was also observed. Notably, the correlation was much stronger when ZJU > 51.27 [*β* = 0.296, 95% CI (0.249, 0.344), *p* < 0.0001] than that when ZJU < 51.27: [*β* = 0.060, 95% CI (0.024, 0.096), *p* = 0.001] (Figure 3 and Table 5).

## Discussion

In the cross-sectional study, ZJU index were positively correlated with hepatic steatosis and liver fibrosis in American adults with NAFLD. Notably, an inverted-L shaped relationship between ZJU index and liver steatosis (inflection point 60.56) was identified. Furthermore, a J-shaped relationship between ZJU index and liver fibrosis was noted, with an inflection point of 51.27. Subgroup analyses revealed a stronger correlation between ZJU index and hepatic steatosis in male subjects or Mexican Americans. Additionally, a stronger correlation between ZJU index and liver fibrosis was evident in male subjects or those with BMI > 30 kg/m^2^.

ZJU index, first introduced by Wang et al. in a cross-sectional study on 9,602 Chinese individuals underwent hepatic ultrasound for NAFLD diagnosis, and now, it has been acknowledged as an effective screening tool for NAFLD in Chinese (AUC = 0.822) (7). Previous studies have confirmed ZJU index’s ability to predict NAFLD in both international and domestic cohorts. In a cross-sectional study on 19,804 Chinese adults, researchers calculated ZJU index, hepatic steatosis index, fatty liver index, visceral adiposity index, and lipid accumulation product for each subject. ZJU index demonstrated high diagnostic accuracy in the identification of NAFLD, with an AUC of 0.925, surpassing the performance of four other indexes. This superior diagnostic capability remained consistent across age and sex subgroups, considering ZJU index as a dependable and effective tool for detecting NAFLD (18). In a separate cohort comprising 28,729 Chinese adults initially without NAFLD, ZJU index was significantly correlated with an increased risk of developing NAFLD over a median follow-up period of 3.01 years. As the quartiles of ZJU index increased, corresponding hazard ratios (HRs) also increased, with values of 4.87 (95%CI = 4.24–5.59) for females and 6.23 (95%CI = 5.56–6.98) for males (8). Additionally, the validation in North Americans was demonstrated in a study on 107 obese females, which reported an AUC of 0.742 for ZJU index, highlighting its utility in predicting NAFLD in this demographic (19). However, existing studies had primarily focused on the correlation between ZJU index and the incidence of NAFLD, without information available on its relationship with CAP and LSM. The primary focus of this investigation was to examine the extent of liver fibrosis and hepatic steatosis in adults with NAFLD in relation to the levels of ZJU. Consequently, this study can be viewed as an expansion and enhancement of prior research efforts.

The degree of liver fibrosis can be evaluated through LSM, while hepatic steatosis can be measured with CAP (20, 21). These findings indicated a notable positive correlation between ZJU and CAP and LSM. Prior studies have shown that the development of fatty liver is linked to impaired functionality of adipose tissues (22), anomalies in glucose processing, and disruptions in lipid metabolism (23, 24). Baseline alterations in BMI have been identified as independent predictors of liver fat (24), with BMI distribution positively associated with the prevalence of NAFLD (25). The components of ZJU index, such as ALT, TG, AST, BMI, and FPG, can be indicative markers for hepatic metabolic abnormalities (26–28). Additionally, a study on 3,329 Chinese adults showed a significant correlation between ZJU index and IR (29). IR is implicated as a contributing risk factor for NAFLD, promoting the progression of fibrosis in nonalcoholic steatohepatitis through various mechanisms including hepatocyte apoptosis, heightened reactive oxygen species production, and disruptions in adipokine and cytokine homeostasis (30).

The findings suggested a notably stronger correlation between ZJU index and hepatic steatosis and liver fibrosis among males rather than females, which may be due to the protective effect of female estrogen on NAFLD (31). Estrogen has been shown to enhance the expression of small heterodimer partner in liver and suppress sterol regulatory element-binding protein 1C (32), thereby inhibiting fatty acid and TG synthesis in liver and potentially ameliorating fatty liver disease (32, 33). Furthermore, estrogen has the potential to modulate the expression of miR-125b through ERα, which in turn suppresses inflammatory injury and fat accumulation in hepatic cells (34). Various epidemiological investigations have suggested a beneficial impact of estrogens on hepatic steatosis in females, aligning with findings from animal studies (35, 36). Surprisingly, there are not any association between ZJU and liver fibrosis in subjects without obesity that have been identified. The degree of liver fibrosis was relatively lower in non-obese participants (37). In addition, non-obese subjects have healthier lifestyles such as exercise and diet compared to obese subjects, which may weaken the association between ZJU and LSM. Furthermore, it was found that the race affected the association between ZJU and CAP. The mechanism of the interaction remains to be determined and could be investigated in the future through basic experiments.

Furthermore, an intriguing discovery of a previously unreported non-linear correlation between ZJU index and hepatic steatosis and liver fibrosis has been made. It is likely that there is a saturating effect of CAP when ZJU index reached 60.56. The correlation between LSM and ZJU index was much stronger when ZJU > 51.27. The findings have the potential to provide new insights into the treatment and prevention for NAFLD.

This study has several strengths. This study represents the initial attempt to investigate the correlation between ZJU index and hepatic steatosis as well as liver fibrosis in individuals with NAFLD by utilizing data from NHANES. This study employed multiple models and incorporates various dimensions, including linear and nonlinear relations, continuous and categorical variables, to support our findings.

This study has several limitations that warrant acknowledgment as well. Firstly, the cross-sectional nature of the study design precluded the establishment of causal relationships. Secondly, the assessment on the severity of liver fibrosis and hepatic steatosis in this study relied on VCTE, a method requiring validation in cohorts with biopsy-proven diagnoses. Additionally, while the data from NHANES was collected from a diverse adult population in the United States, the generalizability of the findings to other geographic regions or ethnic groups may be limited.

The early identification of adult patients at increased risk of advanced hepatic steatosis and fibrosis using non-invasive assessment scores (ZJU) allows early intervention. Hence, we recommend the use of ZJU to risk stratify adults with NAFLD who are at risk of developing advance liver disease.

## Conclusion

In summary, this study showed a significant correlation between ZJU index and the presence of hepatic steatosis and liver fibrosis, indicating its potential utility as a simple biochemical and anthropometric measure for monitoring the severity of NAFLD.

## Data Availability

Publicly available datasets were analyzed in this study. This data can be found here: NHANES, https://www.cdc.gov/nchs/NHANEs/.
